# Diverse parameters of ambulatory knee moments differ with medial knee osteoarthritis severity and are combinable into a severity index

**DOI:** 10.3389/fbioe.2023.1176471

**Published:** 2023-06-13

**Authors:** Baptiste Ulrich, Jennifer C. Erhart-Hledik, Jessica L. Asay, Patrick Omoumi, Thomas P. Andriacchi, Brigitte M. Jolles, Julien Favre

**Affiliations:** ^1^ Swiss BioMotion Lab, Department of Musculoskeletal Medicine, Lausanne University Hospital and University of Lausanne (CHUV-UNIL), Lausanne, Switzerland; ^2^ Department of Orthopaedic Surgery, Stanford University, Stanford, CA, United States; ^3^ Veterans Affairs Palo Alto Health Care System, Palo Alto, CA, United States; ^4^ Department of Mechanical Engineering, Stanford University, Stanford, CA, United States; ^5^ Department of Diagnostic and Interventional Radiology, Lausanne University Hospital and University of Lausanne (CHUV-UNIL), Lausanne, Switzerland; ^6^ Institute of Microengineering, Ecole Polytechnique Fédérale de Lausanne (EPFL), Lausanne, Switzerland; ^7^ The Sense Innovation and Research Center, Lausanne, Switzerland

**Keywords:** gait analysis, kinetics, knee adduction moment, knee flexion moment, biomechanics, machine learning

## Abstract

**Objective:** To characterize ambulatory knee moments with respect to medial knee osteoarthritis (OA) severity comprehensively and to assess the possibility of developing a severity index combining knee moment parameters.

**Methods:** Nine parameters (peak amplitudes) commonly used to quantify three-dimensional knee moments during walking were analyzed for 98 individuals (58.7 ± 9.2 years old, 1.69 ± 0.09 m, 76.9 ± 14.5 kg, 56% female), corresponding to three medial knee osteoarthritis severity groups: non-osteoarthritis (*n* = 22), mild osteoarthritis (*n* = 38) and severe osteoarthritis (*n* = 38). Multinomial logistic regression was used to create a severity index. Comparison and regression analyses were performed with respect to disease severity.

**Results:** Six of the nine moment parameters differed statistically significantly among severity groups (*p* ≤ 0.039) and five reported statistically significant correlation with disease severity (0.23 ≤ |*r*| ≤ 0.59). The proposed severity index was highly reliable (ICC = 0.96) and statistically significantly different between the three groups (*p* < 0.001) as well as correlated with disease severity (*r* = 0.70).

**Conclusion:** While medial knee osteoarthritis research has mostly focused on a few knee moment parameters, this study showed that other parameters differ with disease severity. In particular, it shed light on three parameters frequently disregarded in prior works. Another important finding is the possibility of combining the parameters into a severity index, which opens promising perspectives based on a single figure assessing the knee moments in their entirety. Although the proposed index was shown to be reliable and associated with disease severity, further research will be necessary particularly to assess its validity.

## 1 Introduction

Knee osteoarthritis (OA) is a painful and disabling disease affecting hundreds of millions of people worldwide and this number is expected to grow in the decades to come, notably due to the aging of the population (Wallace et al., 2017; Safari et al., 2020). No cure exists for knee OA and the disease end-stage often leads to major surgery through total knee replacement (Ringdahl and Pandit, 2011), highlighting the need to better understand the pathogenesis of the disease and find ways to slow down its progression.

**FIGURE 1:**
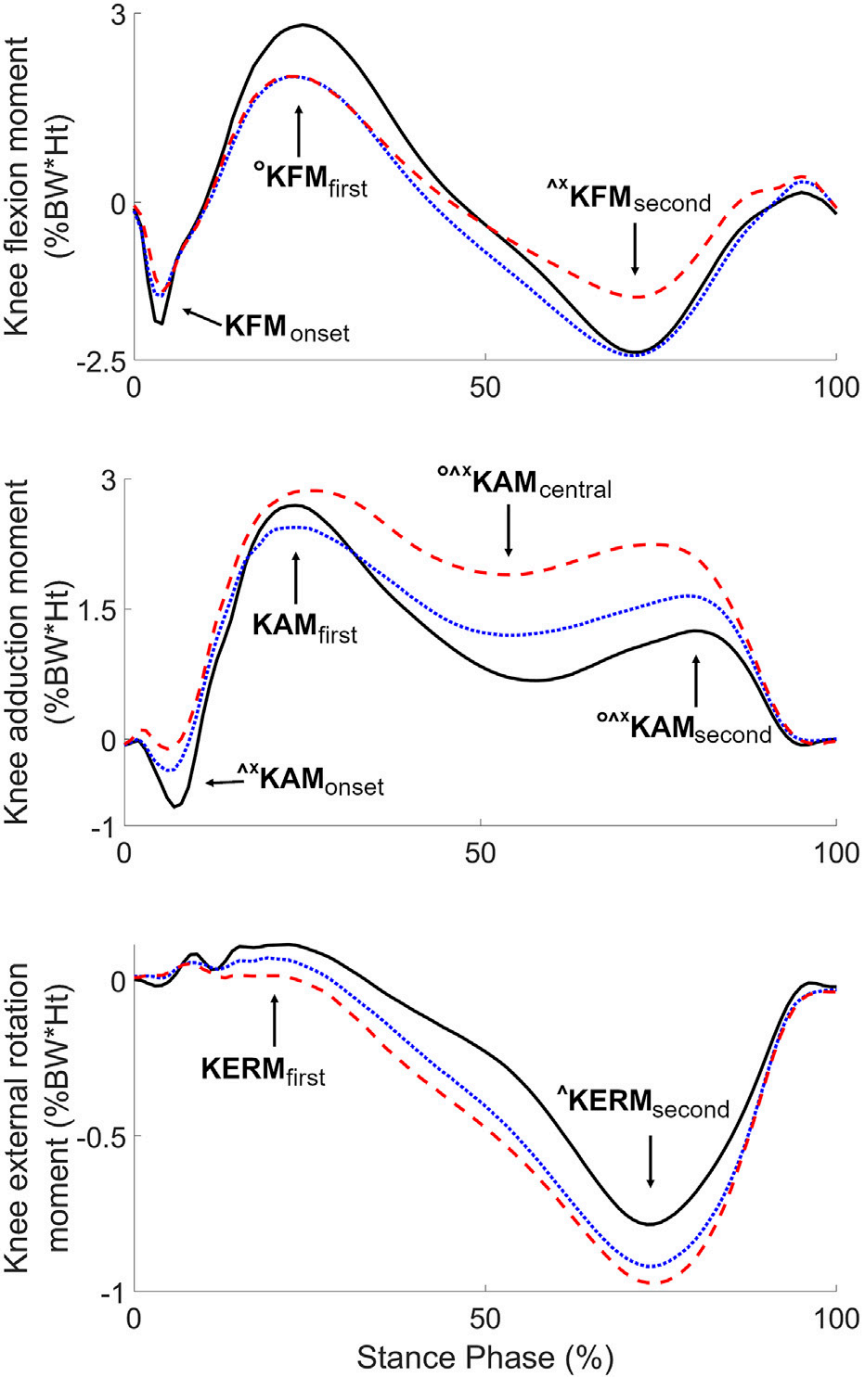
Average knee moments of the three severity groups (black solid lines: non-OA, blue dotted lines: mild OA, red dashed lines: severe OA), with indication of the nine usual parameters. Symbols indicate significant differences between groups (°: non-OA different from mild OA, ^: non-OA different from severe OA, *: mild OA different from severe OA) (*p* < 0.017).

The repetitive mechanical loading at the knee associated with walking has been shown to play an important role in knee OA (Andriacchi et al., 2004). This contribution was particularly well highlighted in a recent report introducing the term “Mechanokine” to stress the unique property of mechanical signals to transcend scales from the external forces acting on the whole-body to the mechanical environment of the cell in a manner that can influence joint heath associated to knee OA (Andriacchi et al., 2020). For instance, the maximum values (peaks) of the knee adduction (KAM_first_) and flexion (KFM_first_) moments during the first half of stance have been related to medial knee OA severity and progression (Kean et al., 2012; Chehab et al., 2014; Erhart-Hledik et al., 2015) and gait modifications based on these parameters showed improvement in clinical outcomes (Cheung et al., 2018; Richards et al., 2018). However, the large majority of previous research on medial knee OA, the most frequent form of the disease (Ahlbäck, 1968), focused on these two parameters and little is known about the seven others usual parameters of knee moments during walking (Figure 1) (Benedetti et al., 1998; Chehab et al., 2017). While analyzing KAM_first_ and KFM_first_ was well motivated in prior works, the disregard of the other parameters was rarely justified. This is even more intriguing that there are evidences scattered across a few specific publications that the other parameters vary with medial knee OA (Thorp et al., 2006; Astephen et al., 2008; Huang et al., 2008; Baert et al., 2013; Mills et al., 2013). Given the possibility that each of the nine parameters illustrated in Figure 1 could influence joint health in different ways and at different stages of the disease, there is a need for comprehensive studies analyzing all nine parameters over the full range of medial knee OA severity.

While considering more parameters will enhance the description of knee moments, having a larger number of parameters to deal with could render the analysis and use of knee kinetics more complex. For example, assessing the effect of a treatment could become difficult when the results diverge among parameters. The situation could be even more arduous with personalized interventions, such as insoles or gait retraining (Reeves and Bowling, 2011), where it could be impossible to find solutions fulfilling modifications on several parameters (Edd et al., 2020; Ulrich et al., 2020). In fact, the increase in complexity when describing knee moments with a higher number of parameters could well be the main reason why most of prior works focused on KAM_first_ and KFM_first_. Therefore, to benefit from a more complete characterization of knee moment without increasing the complexity-of-use, there is a need to combine the parameters into indices associated with specific features of the disease, such as severity. Prior works have already shown the relevance of combining knee moment parameters. For instance, the total joint moment (TJM) combination was introduced to assess the relative contributions of the KAM and KFM (Zabala et al., 2013) and the medial contact force (MCF_first_) parameter to estimate the peak force applied on the medial tibial plateau during the first half of stance (Walter et al., 2010; Manal et al., 2015).

This study first aimed at characterizing all nine usual parameters of knee moments during walking with respect to medial knee OA severity, through comparison and correlation analyses. A second objective was to assess the possibility of developing a severity index combining all nine parameters.

## 2 Methods

### 2.1 Study population

For this study, the database of the Stanford BioMotion lab was screened for individuals aged 40 years old or older, with a body mass index (BMI) lower than 35 kg/m^2^, and who got their gait analyzed following a standard procedure (see below) at the same time they were evaluated for symptoms and imaging signs of knee OA. From those, non-OA individuals, defined as individuals without self-reported pain or significant injury in the lower limb or lower back and without evidence of cartilage loss, osteophytes, subchondral bone marrow lesions, bone attrition, or meniscal pathology (subluxation, maceration, degeneration) in any knees, were selected for the present study (Hunter et al., 2011). Structural alterations of the knees were determined based on magnetic resonance imaging exams, including a three-dimensional fat-suppressed spoiled-gradient recalled echo sequence (3D SPGR; plane = sagittal, TR = 50 ms, TE = 7 ms, flip angle = 30°, field of view = 140 × 140 mm^2^, slice thickness = 1.5 mm, number of slices = 60, acquisition matrix = 256 × 256) and a fat-suppressed proton density fast spin echo sequence (PDFSE; plane = sagittal, TR = 4,000 ms, TE = 13 ms, flip angle = 90°, field of view = 140 × 140 mm^2^, slice thickness = 2.5 mm, number of slices = 33, acquisition matrix = 256 × 256), using a 1.5T machine (GE Medical Systems, Milwaukee, WI). Individuals with medial compartment knee OA were also selected for the present study. These persons were characterized by persistent self-reported pain and radiographic confirmation of the presence of primarily medial compartment OA in at least one knee, no primarily lateral or trochlea OA or arthroplasty in any knees, Kellgren and Lawrence (K/L) grading of both knees (Kellgren and Lawrence, 1957), no diagnosis or symptoms of OA in other lower extremity joints, no serious ankle, hip or back injury or surgery, no gout or recurrent pseudogout, and no use of ambulatory aids. All individuals selected for the present study got their data recorded in the framework of researches approved by the internal review board of Stanford University and gave their consent for further analysis of their data. Data from the most recent testing were used for individuals with multiple records in the database.

In total, 98 individuals (43 males) were available for this study. They were of mean (± standard deviation) age, height and weight of 58.7 ± 9.2 years old, 1.69 ± 0.09 m, and 76.9 ± 14.5 kg, respectively. One knee per individual was analyzed. For non-OA individuals, the study knee was randomly chosen, while the knee with the highest K/L grade was analyzed for OA individuals. In case of equal K/L grade for both knees, the study knee was randomly chosen. For comparison analyses, knees with K/L grade of I or II were considered mild OA and knees with K/L grade of III or IV severe OA, resulting in three severity groups of 22–38 knees each (Table 1). There was no statistically significant demographic difference among the severity groups, except for age, with younger individuals in the non-OA group compared to the two other groups (*p* < 0.001). A *post hoc* power analysis showed that effect sizes of at least 0.8 are detectable with groups of 22–38 knees each (Table 1) considering a power of 80% and a Bonferroni-corrected alpha level of 5% (G*Power, DE). These detectable effect sizes are appropriate, considering the large to strong effect sizes reported in prior studies comparing ambulatory knee moments with respect to OA severity (Cohen’s d between 0.8 and 1.2) (Astephen et al., 2008; Mills et al., 2013).

**TABLE 1:** Characteristics of the three severity groups.

	Non-OA	Mild OA	Severe OA
	*n* = 22	*n* = 38	*n* = 38
Gender (number)	W: 13, M: 9	W: 20, M: 18	W: 22, M: 16
KL grade (number)		I: 26, II: 12	III: 19, IV: 19
Age (years)*	50.5 ± 5.5	59.3 ± 8.6	62.8 ± 8.6
Height (m)	1.70 ± 0.09	1.70 ± 0.10	1.70 ± 0.09
Weight (kg)	74.3 ± 14.7	74.5 ± 14.4	81.0 ± 13.8
Walking speed (m/s)^#^	1.41 ± 0.22	1.28 ± 0.19	1.22 ± 0.19

Data are presented as mean ± SD or as numbers. *Non-OA individuals were statistically significantly younger than mild and severe OA individuals (*p* < 0.001). ^#^Walking speed was statistically significantly faster in non-OA than severe OA individuals (*p* < 0.001).

### 2.2 Gait analysis

All knees in this study were tested following the same standardized procedure including the recording of three 10 m-long straight-line trials at self-selected normal gait speed with personal walking shoes across a walkway instrumented with an optoelectronic motion capture system (Qualisys Medical, Gothenburg, SE) and a force plate (Bertec, Columbs, OH) operating synchronously at 120 Hz. Multiple operators from the same laboratory collected the data. Only trials with a clear step of the foot below the knee of interest on the force plate were recorded. Before recording the gait trials, clusters of reflective markers were fixed on the individuals and a calibration based on anatomical landmarks was performed, following a common protocol (Chehab et al., 2017). During the gait trials, the position and orientation of the lower-limb segments were calculated using the cluster marker trajectories and the calibration information (Andriacchi et al., 1998; Favre et al., 2009). The flexion, adduction and external rotation moments at the knee during the stance phases with the foot of interest on the force plate were calculated following a standard inverse dynamics approach (Zabala et al., 2013). The three moments were time-normalized to 0%–100% during stance and expressed as external moments in percentage of bodyweight and height (%BW × Ht). During each stance phase, the nine characteristic parameters of the moment curves were extracted for analysis (Table 2; Figure 1) (Benedetti et al., 1998; Chehab et al., 2017). Finally, each of the nine parameters was averaged over the three trials to have one value per parameter and knee. All biomechanical processing was done using the software application BioMove (Stanford, CA).

**TABLE 2:** Values of the knee moment parameters for the three severity groups, as well as Spearman correlation between the parameters and disease severity.

Parameter	Definition	Values per severity group: median (1st quartile; 3rd quartile)			Correlation with disease severity, *n* = 98			
		Non-OA, *n* = 22	Mild OA, *n* = 38	Severe OA, *n* = 38	Spearman correlation		Partial spearman correlation^Δ^	
					r_s_ (95% CI)	*p*-value	r_s_ (95% CI)	*p*-value
KAM_central_	Minimum adduction moment between KAM_first_ and KAM_second_	0.61 (0.30; 0.86)^#§^	0.98 (0.77; 1.56)*^§^	1.69 (1.17; 2.55)*^#^	0.59 (0.43; 0.71)	<0.001	0.52 (0.35; 0.66)	<0.001
KFM_second_	Minimum flexion moment during second half of stance	−2.34 (−2.92; −1.87)^§^	−2.58 (−3.15; −1.87)^§^	−1.56 (−2.38; −0.79)*^#^	0.35 (0.16; 0.52)	<0.001	0.21 (0.01; 0.40)	0.037
KAM_first_	Maximum adduction moment during first half of stance	3.01 (2.34; 3.24)	2.49 (2.16; 3.22)	3.16 (2.50; 3.82)	0.13 (−0.07; 0.32)	0.189	0.31 (0.11; 0.48)	0.002
KERM_second_	Minimum external rotation moment during second half of stance	−0.79 (−1.00; −0.70)^§^	−0.94 (−1.07; −0.74)	−0.96 (−1.19; −0.76)*	−0.24 (−0.42; −0.04)	0.016	−0.36 (−0.53; −0.17)	<0.001
KFM_first_	Maximum flexion moment during first half of stance	3.00 (2.21; 3.61)^#^	2.06 (1.47; 2.52)*	2.05 (0.85; 3.43)	−0.17 (−0.36; 0.03)	0.095	−0.01 (−0.21; 0.18)	0.893
KAM_onset_	Minimum adduction moment before KAM_first_	−0.87 (−0.55; −1.19)^§^	−0.83 (−0.40; −0.94)^§^	−0.37 (−0.13; −0.54)*^#^	0.48 (0.30; 0.63)	<0.001	0.46 (0.28; 0.61)	<0.001
KAM_second_	Maximum adduction moment during second half of stance	1.32 (0.91; 1.69)^#§^	1.62 (1.40; 2.05)* ^§^	2.40 (1.64; 2.89)*^#^	0.49 (0.31; 0.63)	<0.001	0.53 (0.35; 0.66)	<0.001
KERM_first_	Maximum external rotation moment during first half of stance	0.19 (0.11; 0.26)	0.16 (0.11; 0.22)	0.12 (0.07; 0.19)	−0.23 (−0.42; −0.03)	0.021	−0.11 (−0.31; 0.09)	0.273
KFM_onset_	Minimum flexion moment before KFM_first_	−2.42 (−2.93; −1.68)	−2.08 (−2.49; −1.60)	−1.88 (−2.33; −1.64]	0.18 (−0.02; 0.37)	0.077	0.04 (−0.16; 0.23)	0.711

All parameters are reported in %BW*Ht. See Figure 1 for an illustration of the parameters and their differences with disease severity. To facilitate reading across tables, parameters are reported following the order in Table 4. ^Δ^Partial correlation between knee moment parameters and disease severity while controlling for age and walking speed. *significantly different compared to the non-OA group (*p* < 0.017). ^#^significantly different compared to the mild OA group (*p* < 0.017). ^§^significantly different compared to the severe OA group (*p* < 0.017).

### 2.3 Severity index

The severity index was computed by multinomial logistic regression with the severity groups as nominal response and the nine knee moment parameters as predictors (McCullagh and Nelder, 2019). The parameters were standardized using a z-score transformation before performing the regression and a sigmoid transformation was applied to the regressed data to have indices ranging between 0 and 100. The regression was calculated by bootstrapping which allowed determining confidence intervals for the regression coefficients and assessing the reliability of the index (Efron and Tibshirani, 1994). Reliability was characterized using the intraclass correlation coefficient (ICC) and the standard error of measurement (SEM) (Weir, 2005).

For completeness with literature, two previously proposed combinations of knee moment parameters, the total knee joint moment (Zabala et al., 2013) and the medial contact force (Walter et al., 2010; Manal et al., 2015), were also computed. For the total knee joint moment, the square root of the sum of the squared knee flexion, adduction and external rotation moments was calculated for each time point of each stance (Zabala et al., 2013). Then, the maximal values during the first and second halves of each stance were extracted (TJM_first_ and TJM_second_, respectively). Regarding the medial contact force, the maximum value during the first half of each stance (MCF_first_) was estimated based on KAM_first_ and KFM_first_ using a formula determined with instrumented knee prostheses (Walter et al., 2010; Manal et al., 2015; Uhlrich et al., 2018). Similar to the other moment parameters, TJM_first_, TJM_second_ and MCF_first_ were averaged over the three trials recoded for each knee.

### 2.4 Statistical analysis

Since the data were not normally distributed (Kolmogorov-Smirnov tests), they were analyzed using non-parametric statistics. Specifically, comparisons of the nine knee moment parameters, the proposed severity index, the three prior combination parameters and the walking speed among the severity groups were performed using Kruskal-Wallis tests with *post hoc* ranksum tests. Associations with disease severity, for the knee moment parameters, the severity index and the prior combination parameters, were assessed using Spearman correlations across severity groups. Since walking speed and age have been shown to influence knee moment parameters (Lelas et al., 2003; Chehab et al., 2017), partial Spearman correlations were also calculated to describe the relationship with disease severity while controlling for walking speed and age. Finally, Spearman correlations were performed to quantify the associations among parameters. Significance level was set *a priori* at *p* < 0.05, with Bonferroni corrections for multiple comparisons during *post hoc* analyses (effective *p* < 0.017).

## 3 Results

Six of the nine knee moment parameters showed statistically significant effect of disease severity (KFM_first_, *p* = 0.034; KERM_second_, *p* = 0.039; KAM_central_, KFM_second_, KAM_onset_, and KAM_second_, all *p* < 0.001). Post-hoc testing indicated significant incremental differences in KAM_central_ and KAM_second_ from non-OA to mild OA, to severe OA, with larger values in more severely affected knees (Table 2; Figure 1). KAM_onset_ was significantly larger in the severe OA group compared to both the non-OA and the mild OA groups. KFM_second_ was significantly larger in severe OA knees compared to both the non-OA and the mild OA knees, while KFM_first_ was significantly smaller in mild OA than in non-OA knees. Finally, KERM_second_ was significantly smaller in the severe OA than in the non-OA group.

Statistically significant correlations with disease severity were found for five of the nine moment parameters: KAM_central_ (r_s_ = 0.59, *p* < 0.001), KAM_second_ (r_s_ = 0.49, *p* < 0.001), KAM_onset_ (r_s_ = 0.48, *p* < 0.001), KFM_second_ (r_s_ = 0.35, *p* < 0.001), KERM_second_ (r_s_ = −0.24, *p* = 0.016) and KERM_first_ (r_s_ = −0.23, *p* = 0.021) (Table 2). Controlling for age and walking speed resulted in the same statistically significant correlations, except for KAM_first_ which became significant (r_s_ = 0.31, *p* = 0.002) and KERM_first_ which exceeded the significance level (r_s_ = −0.11, *p* = 0.273). Correlations among the nine moment parameters are reported in Supplementary Table S1.

The proposed regression method allowed compiling a severity index showing an excellent reliability, with ICC of 0.96 and SEM of 6.78 units (Koo and Li, 2016). Moreover, Kruskal-Wallis test showed statistically significant differences among severity groups (*p* < 0.001), with *post hoc* analyses indicating significant difference between the three groups. Indeed, the non-OA group had significantly lower severity indices than the mild OA and severe OA groups, and the mild OA group had significantly lower severity indices than the severe OA groups (Table 3). Additionally, a significant correlation was found between the severity index and disease severity (r_s_ = 0.70, *p* < 0.001). The correlation remained significant when controlling for age and walking speed (r_s_ = 0.63, *p* < 0.001).

**TABLE 3:** Values of the severity index and of three prior moment combination parameters for the three severity groups, as well as Spearman correlation between these measures and disease severity.

Combination parameter	Definition; unit	Values per severity group: median (1st quartile; 3rd quartile)			Correlation with disease severity, *n* = 98			
		Non-OA, *n* = 22	Mild OA, *n* = 38	Severe OA, *n* = 38	Spearman correlation		Partial spearman correlation^Δ^	
					r_s_ (95% CI)	*p*-value	r_s_ (95% CI)	*p*-value
Severity index	Severity index; -	10.0 (3.7; 21.0)^#^^§^	42.9 (25.3; 58.2)*^§^	84.2 (62.5; 94.4)*^#^	0.70 (0.57; 0.80)	<0.001	0.63 (0.48; 0.75)	<0.001
TJM_first_	Maximum total knee joint moment during first half of stance; %BW*Ht	4.11 (3.53; 4.75)	3.29 (2.90; 4.25)	3.95 (3.20; 4.95)	0.01 (−0.19; 0.21)	0.893	0.31 (0.11; 0.48)	0.002
TJM_second_	Maximum total knee joint moment during second half of stance; %BW*Ht	2.72 (2.27; 3.01)	3.27 (2.42; 3.82)	3.06 (2.72; 3.46)	0.11 (−0.09; 0.30)	0.277	0.25 (0.05; 0.43)	0.014
MCF_first_	Maximum medial contact force during first half of stance; BW	2.07 (1.90; 2.27)	1.87 (1.72; 2.08)	2.12 (1.79; 2.25)	0.03 (−0.16; 0.23)	0.734	0.63 (0.48; 0.75)	0.004

^Δ^partial correlation between knee moment parameters and disease severity while controlling for age and walking speed. *significantly different compared to the non-OA group (*p* < 0.017). ^#^significantly different compared to the mild OA group (*p* < 0.017). ^§^significantly different compared to the severe OA group (*p* < 0.017).

**FIGURE 2:**
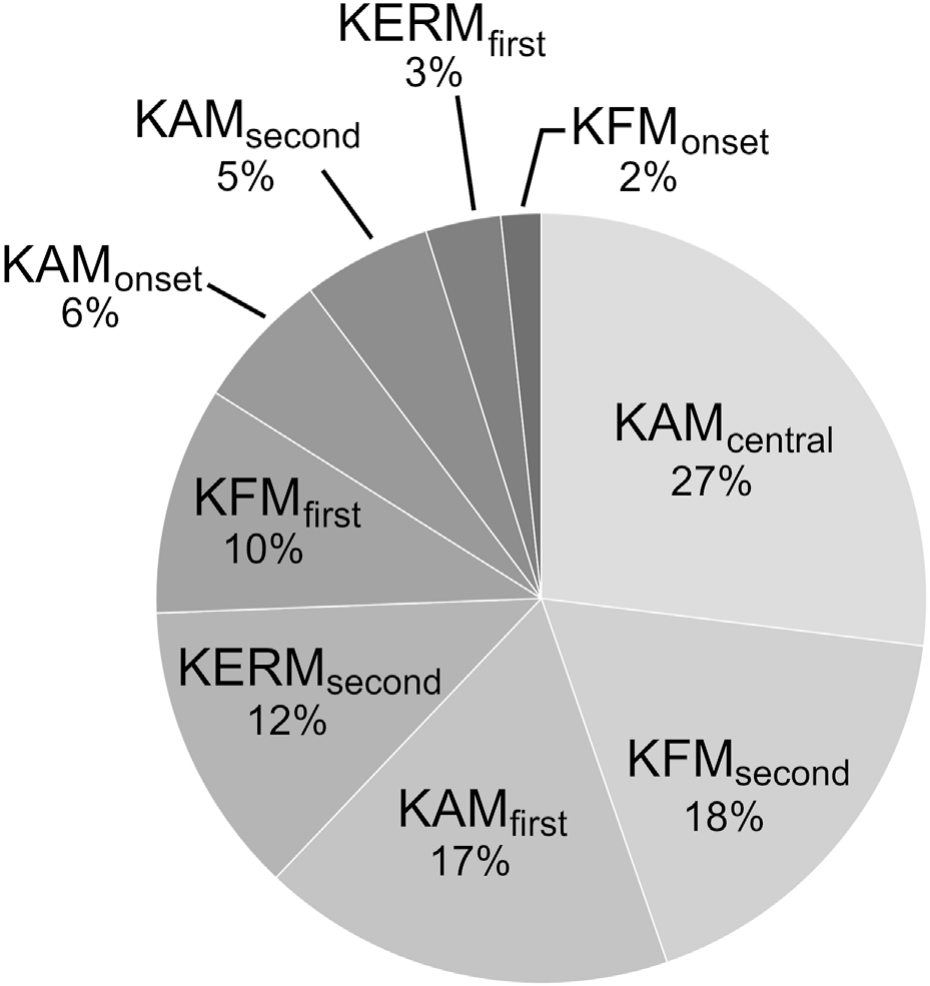
Contribution of the nine moment parameters to the severity index.

Since the moment parameters were standardized before calculating the severity index, the coefficients of the regression leading to the severity index can be analyzed to compare the contribution of the nine moment parameters to the severity index. Doing so, indicated that KAM_central_ had the biggest effect on the index with a coefficient of −1.52, contributing 27.0% to the severity index, followed by KFM_second_ and KAM_first_ with coefficients of −1.00 and 0.98 and contributing for 17.7% and 17.4% of the severity index, respectively (Table 4; Figure 2). On the opposite, KFM_onset_, KERM_first_ and KAM_second_ had the least impact on the index, with coefficients of 0.095, −0.178, and −0.304 and contributions of 1.7%, 3.2% and 5.4% to the severity index, respectively.

**TABLE 4:** Coefficient of the nine moment parameters in the severity index regression.

Parameter	Regression coefficient (95% CI)
KAM_central_	−1.52 (−1.58; −1.47)
KFM_second_	−1.00 (−1.03; −0.97)
KAM_first_	0.98 (0.94; 1.02)
KERM_second_	0.69 (0.65; 0.74)
KFM_first_	0.54 (0.51; 0.57)
KAM_onset_	−0.33 (−0.37; −0.28)
KAM_second_	−0.30 (−0.37; −0.24)
KERM_first_	−0.18 (−0.21; −0.15)
KFM_onset_	0.09 (0.07; 0.12)

Parameters are ordered according to the magnitude of the regression coefficients. Please refer to Table 2 and Figure 1 for a definition and an illustration of the parameters, respectively.

Additionally, Kruskal-Wallis tests showed no statistically significant difference among severity groups for any of the three prior combinations parameters (TJM_first_, *p* = 0.079; TJM_second_, *p* = 0.168; MCF_first_, *p* = 0.061). When controlling for age and walking speed, statistically significant correlations with disease severity were observed for all three combinations: TJM_first_ (r_s_ = 0.31, *p* = 0.002), TJM_second_ (r_s_ = 0.25, *p* = 0.014), and MCF_first_ (r_s_ = 0.29, *p* = 0.004). The correlations were non-significant when no control for age and walking speed was applied (r_s_ ≤ 0.13, *p* ≥ 0.188).

## 4 Discussion

This study confirmed that diverse knee moment parameters differ with respect to the severity of medial knee OA. Compared to prior works, the present study, testing all usual parameters over the entire spectrum of disease severity, provided a basis to assemble the pieces disseminated in literature. Various factors, including participants’ characteristics or analysis protocols, could influence knee moments and lead to diverging results among studies (Messier et al., 2005; Chehab et al., 2017; Schrijvers et al., 2021). Nevertheless, even with such possible methodological variations among studies, strong consensuses could be identified for four parameters. These included smaller KFM_first_ in mild OA compared to non-OA (asymptomatic) knees and in severe OA compared to mild OA knees, although the severe-mild difference was not observed in the present study (Weidow et al., 2006; Astephen et al., 2008; Huang et al., 2008). Consistent observations also existed for larger KFM_second_ in severe OA than in non-OA (asymptomatic) and mild OA knees (Astephen et al., 2008; Baert et al., 2013), as well as larger KAM_central_ and KAM_second_ in mild OA than in non-OA (asymptomatic) and in severe OA than in mild OA (Thorp et al., 2006; Astephen et al., 2008; Huang et al., 2008). No consensus existed for KAM_first_, which was already shown to have highly inconsistent results among studies (Mills et al., 2013), and no consolidation could be attempted for the other parameters due to lacking data in literature. Altogether, the present study shed light on three parameters, KAM_central_, KAM_second_, and KFM_second_, which were frequently disregarded in prior works. This suggests that future research should not limit the analysis to KAM_first_ and KFM_first_. This suggestion is particularly well supported by two recent studies relating KAM_central_ with disease progression and symptoms (Astephen Wilson et al., 2017; Costello et al., 2020).

With the consideration of more than two parameters appearing wise for the characterization of the knee moments, the possibility to combine the parameters into an index reflecting disease severity constitutes another important finding of the present study. Indeed, while considering a larger number of parameters will contribute to better descriptions, having a larger number of parameters to manage could increase study design complexity and make gait interventions more complex (Edd et al., 2020). Therefore, the possibility of combining the parameters, as demonstrated in this study, is interesting practically. However, beyond practical considerations, indices could be especially relevant for the global assessments of the knee moments they allow. For example, in personalized interventions, such as gait retraining (Cheung et al., 2018; Richards et al., 2018; Ulrich et al., 2020), it could become possible to aim for a global change, instead of aiming for changes in one or two moment parameters, without consideration for the others.

The second objective of assessing the possibility of developing a severity index was fully achieved, with the design of an index reliable, significantly different among the three severity groups and showing a large correlation with disease severity. Further research will now be necessary to assess the validity of the proposed index. The techniques to record and calculate knee moments differ among institutions (Benedetti et al., 2013; Schrijvers et al., 2021). Therefore, the sensitivity of the severity index to variations in gait analysis protocols will need to be determined. It is well possible that the index will be little sensitive to such methodological differences, as it is an aggregate of standardized parameters. It will also be necessary evaluating the index longitudinally and characterizing its relationships with key features of knee OA, such as pain or disease progression (Felson, 2009).

It is interesting to note that KAM_first_ accounted for 17% of the severity index (third most important contributor to the index), although it was not significantly different among severity groups. While such an important role in the severity index could appear peculiar in view of its relationship with disease severity, this role well agrees with medial knee OA literature, where KAM_first_ is a prevalent parameter and the primary focus of gait interventions (Reeves and Bowling, 2011; Mills et al., 2013; Favre and Jolles, 2016). Thus, it is possible that the severity index actually captured the global essence of disease severity. Three combination parameters were already proposed in literature, TJM_first_, TJM_second_, and MCF_first_ (Walter et al., 2010; Zabala et al., 2013; Manal et al., 2015), but the severity index in this study is the first to have been designed to reflect disease severity.

The present study brought new insights into the relationship between knee moment parameters and disease severity that could reveal particularly useful in the evaluation and rehabilitation of medial knee OA gait (Favre and Jolles, 2016). Nevertheless, further research will be necessary to determine the mechanisms behind these relationships. Walking speed and age certainly play a role in the relationships between knee moment parameters and disease severity, but, as confirmed in this study, the causes are more complex than simply variations in walking speed or in age (Landry et al., 2007). Consequently, the role of other factors, including motor control, muscle strength and soft-tissue properties (Lewek et al., 2004; Hubley-Kozey et al., 2006; Rudolph et al., 2007; Adouni and Shirazi-Adi, 2014; Stanahan et al., 2015) as well as pain (Boyer, 2018), will need to be clarified in future studies. Further works should also assess the relevance of the severity index in pre-OA both for early disease detection and gait modification (Reeves and Bowling, 2011; Ulrich et al., 2020).

This study has some limitations, including the use of a single cross-sectional dataset, as discussed above. Multiple operators contributed to the gait data collection, which could have led to increased inter-individual variability and limited the detection of differences and correlations with disease severity. Nevertheless, obtaining conclusive results based on data collected by multiple operators remained a strength in view of future large-scale applications where gait recording will likely be performed by different operators. Another point worth mentioning is the multinomial logistic regression used to determine the severity index. While it is a common method, which successfully combined the moment parameters, one cannot exclude that there could be other ways to combine the parameters. This is particularly supported by the fact that some parameters were correlated to each other. It is important to note that the possible existence of alternative combinations does not affect the main findings but requires caution to not over interpret the combination obtained in this study as unique or being the best. Depending on the results of the validity studies to follow, in the future, it might be necessary comparing different combination methods. Additionally, in line with literature, this study focused on discrete knee moment parameters. Nevertheless, analyzing the knee moment curves could also reveal interesting, for example, using one-dimensional statistical parametric mapping (Friston et al., 1994; Pataky, 2012). Finally, since the severity groups differed in age and walking speed, it is possible that a fraction of the severity index reflects the variations in moment parameters with respect to age or walking speed.

## 5 Conclusion

This study confirmed that diverse knee moment parameters differ with disease severity. In particular, differences among severity groups were found to be consistent across studies for four parameters, including three that were frequently disregarded in prior works (KAM_central_, KAM_second_, KFM_second_). Future studies are therefore recommended to not limit the analysis to KAM_first_ and KFM_first_. Another important finding of this study was the possibility to combine the parameters into a severity index, which opens promising perspectives based on a single figure assessing the knee moments in their entirety. While the proposed index was shown to be reliable and correlated with disease severity, further research will be necessary to assess its validity.

## Data Availability

The data analyzed in this study is subject to the following licenses/restrictions: The data are not publicly available due to regulatory provisions. Requests to access these datasets should be directed to baptiste.ulrich@chuv.ch.
