# Supplementary material for: Diverse parameters of ambulatory knee moments differ with medial knee osteoarthritis severity and are combinable into a severity index
Source: Front Bioeng Biotechnol. 2023 Jun 13;11:1176471. doi: 10.3389/fbioe.2023.1176471 (PMC10293674; doi:10.3389/fbioe.2023.1176471)
Supplement: Supplementary file 1 [file Table1.DOCX]

**Supplementary Table 1:** Spearman’s correlation matrix for the nine moment parameters.

|  | **KFM_second_** | **KAM_first_** | **KERM_second_** | **KFM_first_** | **KAM_onset_** | **KAM_second_** | **KERM_first_** | **KFM_onset_** |
| --- | --- | --- | --- | --- | --- | --- | --- | --- |
| **KAM_central_** | 0.20* | 0.48*** | -0.52*** | -0.27** | 0.53*** | 0.79*** | -0.30** | 0.18 |
| **KFM_second_** |  | 0.01 | 0.17* | 0.39*** | 0.36*** | 0.15 | -0.07 | 0.29*** |
| **KAM_first_** |  |  | -0.67*** | 0.07 | 0.30** | 0.66*** | -0.07 | -0.15 |
| **KERM_second_** |  |  |  | 0.04 | -0.35*** | -0.73*** | 0.09 | 0.09 |
| **KFM_first_** |  |  |  |  | 0.02 | -0.05 | 0.49*** | 0.18 |
| **KAM_onset_** |  |  |  |  |  | 0.54*** | -0.22* | 0.25** |
| **KAM_second_** |  |  |  |  |  |  | -0.12 | 0.02 |
| **KERM_first_** |  |  |  |  |  |  |  | -0.04 |

* p < 0.05

** p < 0.01

*** p < 0.001
